# Apathy Assessment in Neurocognitive Disorders: Revisiting a Supportive Clinical Feature for the Early Recognition of Prodromal Dementia With Lewy Bodies

**DOI:** 10.1111/psyg.70214

**Published:** 2026-09-02

**Authors:** Hiroshige Fujishiro, Kuniyuki Iwata, Hideki Kanemoto, Madoka Yanagawa, Hajime Baba

**Affiliations:** ^1^ Department of Psychiatry Nagoya University Graduate School of Medicine Nagoya Japan; ^2^ Health and Counseling Center The University of Osaka Toyonaka Osaka Japan; ^3^ Department of Clinical Oncology and Chemotherapy Nagoya University Graduate School of Medicine Nagoya Japan; ^4^ Department of Psychiatry Juntendo University Koshigaya Hospital Koshigaya Japan

With the advent of disease‐modifying therapies for neurocognitive disorder (NCD), particularly Alzheimer's disease (AD), accurate early differential diagnosis based on the underlying neuropathology has become increasingly essential. However, clinicopathological studies have consistently demonstrated the substantial diagnostic challenges involved in distinguishing AD from dementia with Lewy bodies (DLB), the second most common NCD, especially during the prodromal stage. Analyses from the National Alzheimer's Coordinating Center (NACC) have shown that clinical diagnoses of AD frequently fail to detect underlying Lewy body disease (LBD), a neuropathological entity encompassing three pathological subtypes: brainstem‐predominant, transitional, and diffuse neocortical LBD [1]. Among individuals clinically diagnosed with AD at the Clinical Dementia Rating (CDR) 0.5 stage, approximately one‐third were found to have LBD. These findings highlight the need for additional clinical markers that may facilitate differentiation between prodromal DLB and AD during the earliest symptomatic phases.

Although diagnostic approaches have traditionally focused on cognitive impairment, neuropsychiatric symptoms are increasingly recognised as early manifestations of NCDs. In this context, the construct of Mild Behavioral Impairment (MBI) provides a useful framework for understanding the clinical significance of later‐life behavioural changes [2]. Within the five MBI domains, decreased motivation (apathy) has been identified as one of the most prevalent behavioural syndromes. Longitudinal cohort studies have demonstrated that persistent apathy in older adults is associated with an increased risk of incident dementia, even among individuals with minimal cognitive impairment [3]. These observations emphasise the clinical relevance of systematically assessing motivational changes during the prodromal stage and suggest that apathy may serve as an early behavioural indicator of neurodegenerative pathology.

Despite this growing recognition, apathy has received relatively limited attention as a feature of prodromal DLB. Accumulating evidence suggests that apathy may represent an early behavioural manifestation of LBD [4]. From a pathophysiological perspective, apathy in DLB is thought to reflect dysfunction within distributed motivational networks involving frontal‐subcortical and limbic circuits, including the frontal cortex, anterior cingulate cortex, and ventral striatum. Although β‐amyloid deposition and neurofibrillary tangles are commonly observed in these regions of both AD and DLB, dopaminergic degeneration associated with LBD is neuropathologically specific to DLB. The nigrostriatal and mesocorticolimbic dopaminergic pathways are already evident during prodromal DLB and may contribute to reduced reward sensitivity, diminished initiative, and impaired goal‐directed behaviour. Current diagnostic frameworks for prodromal DLB primarily emphasise mild cognitive impairment associated with Lewy bodies (MCI‐LB) as a major clinical presentation, within which apathy has been recognised as a supportive clinical feature [5]. In contrast, apathy in AD appears to increase progressively with disease severity, being reported in approximately 19% of mild AD cases and up to 88% of moderate‐to‐severe cases [6]. These findings raise the possibility that prominent apathy during the prodromal stage may be more characteristic of DLB than of early AD.

Recent clinical studies further support this interpretation. A systematic review reported pooled apathy prevalence of approximately 46% in MCI‐LB and 57% in DLB [6]. In more recent investigations, LBD‐related clinical features were comprehensively assessed, and a subset of participants underwent neuropathological confirmation of LBD [6]. Across these studies, apathy was observed in approximately 50%–70% of individuals with prodromal DLB, whereas prevalence estimates in prodromal AD generally remained below 20% [6]. Although methodological differences among studies should be acknowledged, the consistency of these findings suggests that apathy may provide a clinically meaningful behavioural signal supporting differentiation between underlying pathologies during the prodromal stage. Nevertheless, confirmation through large prospective longitudinal studies incorporating AD and DLB biomarkers remains necessary.

Methodological considerations regarding the assessment of apathy are also crucial. Many previous studies have relied on the Neuropsychiatric Inventory (NPI), which was originally developed to evaluate behavioural symptoms in individuals with established dementia. Although widely used, the NPI was designed primarily to quantify symptom burden rather than to establish syndrome‐specific diagnoses. In contrast, the recently proposed diagnostic criteria for apathy in NCD emphasise persistent changes in initiative, interest, and emotional expression relative to premorbid functioning [7]. Adoption of these revised criteria may improve recognition of clinically meaningful apathy and facilitate earlier identification of underlying NCD in routine clinical practice. Here, we proposed a six‐step clinical approach to identifying apathy based on current criteria in NCD (Figure 1) [7]. This framework may facilitate investigation into the accurate prevalence of apathy at prodromal stages of various NCDs. Further studies with pathological verification are needed to clarify their clinical applicability and research utility.

**FIGURE 1:**
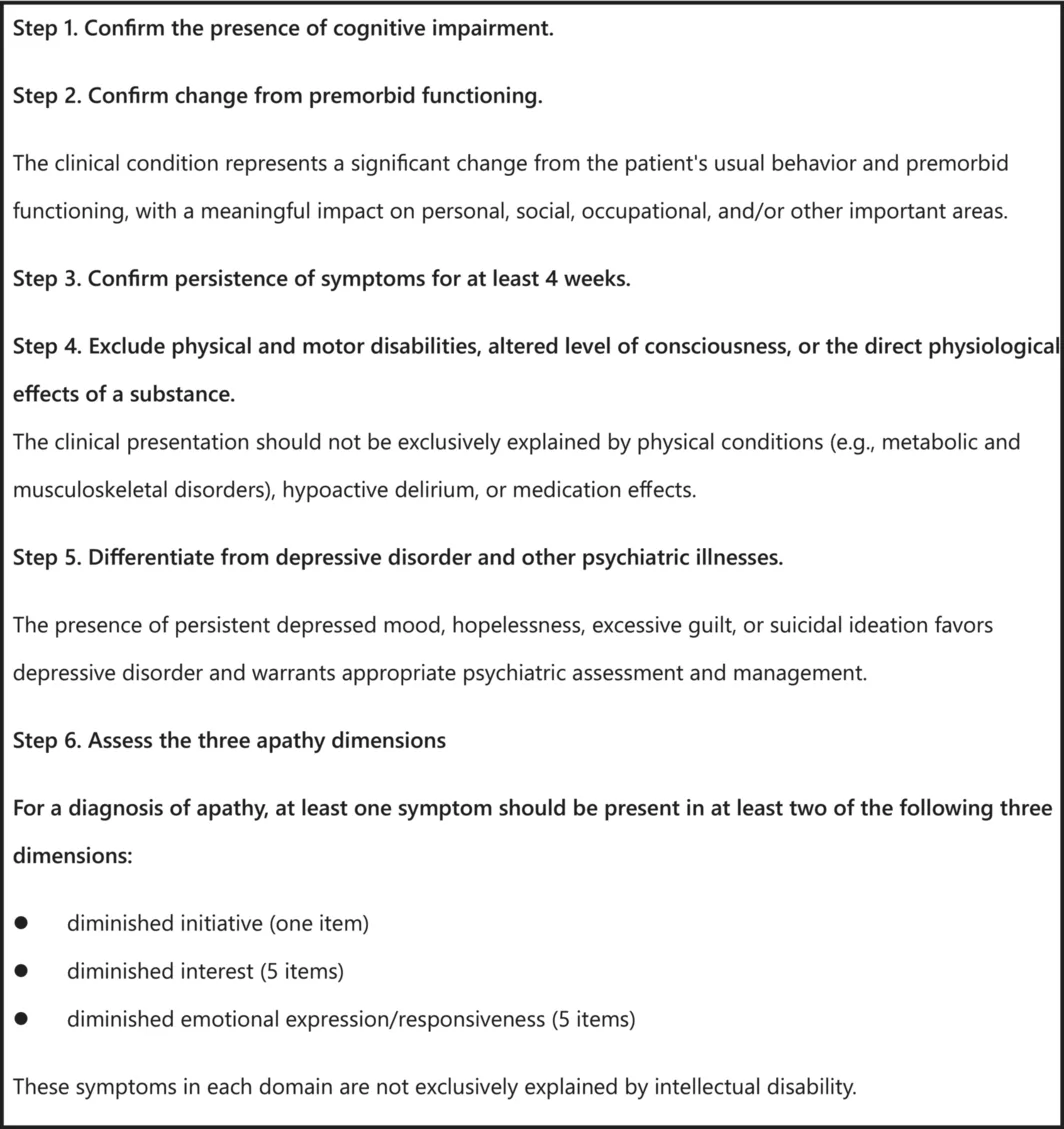
A proposed six‐step clinical approach for identifying apathy in patients with neurocognitive disorder based on current diagnostic criteria [7].

In summary, accumulating evidence indicates that apathy is highly prevalent during the prodromal stages of DLB and occurs more frequently than in prodromal AD. Nevertheless, apathy has not yet been systematically incorporated into routine differential diagnostic assessment. Given the substantial underrecognition of LBD among individuals clinically diagnosed with early AD, careful assessment of apathy based on the revised diagnostic criteria for NCD may provide a clue to diagnose prodromal DLB [7]. This approach should be further explored through future studies incorporating biomarkers and neuropathological analyses.

## Funding

This work was supported by the Japan Agency for Medical Research and Development (JP25dk0110053).

## Conflicts of Interest

The authors declare no conflicts of interest.

## Data Availability

Data sharing not applicable to this article as no datasets were generated or analysed during the current study.
